# Does the Gut Microbiota Contribute to Obesity? Going beyond the Gut Feeling

**DOI:** 10.3390/microorganisms3020213

**Published:** 2015-04-27

**Authors:** Marisol Aguirre, Koen Venema

**Affiliations:** 1 Top Institute of Food and Nutrition, P.O. Box 557, 6700 AA Wageningen, The Netherlands; E-Mail: Marisol.aguirremorales@tno.nl; 2 School of Nutrition and Translational Research in Metabolism (NUTRIM), Faculty of Health, Medicine and Life Sciences, Department of Human Biology, Maastricht University, P.O. Box 616, 6200 MD Maastricht, The Netherlands; 3 The Netherlands Organization for Applied Scientific Research (TNO), P.O. Box 360, 3700 AJ Zeist, The Netherlands; 4 Beneficial Microbes Consultancy, Johan Karschstraat 3, 6709 TN Wageningen, The Netherlands

**Keywords:** obesity, gut microbiota, microbial ecology, energy balance, metabolism

## Abstract

Increasing evidence suggests that gut microbiota is an environmental factor that plays a crucial role in obesity. However, the aetiology of obesity is rather complex and depends on different factors. Furthermore, there is a lack of consensus about the exact role that this microbial community plays in the host. The aim of this review is to present evidence about what has been characterized, compositionally and functionally, as obese gut microbiota. In addition, the different reasons explaining the so-far unclear role are discussed considering evidence from *in vitro*, animal and human studies.

## 1. Introduction

The adoption of a modern/Western type lifestyle, characterized by a high consumption of energy-dense foods and reduced physical activity, has been accompanied by the growth of obesity in developed and industrialized countries [1,2]. Still, the long-held belief of considering obesity as mainly associated with an imbalance in energy consumed when compared to energy expenditure, seems to be incomplete given the recent mechanisms proposed to underlie obesity [1]. Namely, growing evidence suggests a less simplistic event which involves a combination of factors including: environment, genetics, diet and lifestyle, adipose tissue and systemic inflammation [1,3,4]. Moreover, the gut microbiota has been proposed as an environmental factor that plays a crucial role in obesity [1].

It is estimated that the bacterial cells in and on the body outnumber by 10 times the amount of human cells [5]. In particular, the human gastrointestinal tract hosts approximately 10^14^ microbes which are mostly prevalent in the large intestine [5]. Not surprisingly, the gut microbiota has been studied the most in adults [6]. It consists of a community of primarily *Firmicutes*, *Bacteroidetes*, *Actinobacteria*, *Fusobacteria*, *Proteobacteria* and *Verrucomicrobia* [6]. Approximately 200–300 species are part of such dense community in an individual (estimated to contain around 10^12^ cells per gram) [7]. The combination of the proteins/enzymes encoded by their genomes (more than 5 million genes) yields additional molecules and grants special functions exceeding the host’s own genetic potential by two orders of magnitude [7,8]. Interesting examples about the influence of the metabolic activity of the microbiota on humans include the observations by Hehemann *et al.* [9], who identified β-porphyranase, an enzyme previously found in the seaweed-associated bacterium *Zobellia glactanivorans*, in the genome of *Bacteroides plebeius*, a bacterium that has been only isolated in Japanese individuals. Strikingly, β-porphyranase confers the capacity to hydrolyze indigestible polysaccharides present in marine plants. Another interesting example includes the study in which it was found that African children could digest cellulose due to their unique gut microbiota [10].

**Figure 1:**
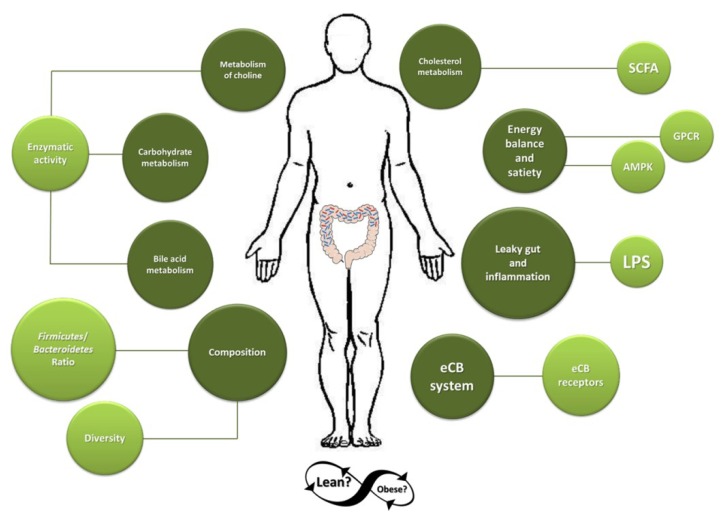
The gut microbiota may lead to obesity by disturbing host homeostasis.

Obesity, in terms of microbiota, is a complicated disequilibrium that presents many complications (Figure 1). Chronic low-grade endotoxemia, modulation of secretion of gut-derived peptide hormones, regulation of active adipose tissue composition and increased energy harvest from host diet have been suggested as mechanisms through which the gut microbiota may contribute to obesity [11]. Still, the role of the gut microbiota in human obesity remains unclear. Different reasons may explain this lack of consensus. On the one hand, most studies have been done in rodent models which carry some disadvantages due to the differences in terms of gut microbiota composition, fermentation process (location, rates of digesta passage, *etc.*) and dietary practices (coprophagia) [12,13]. On the other hand, studies in humans have shown a large inter-individual variation in the gut microbiota composition and the different methods used to analyze the bacteria together with involving participants with different backgrounds (food habits and ethnicity) constitute factors that influence the sometimes contradictory results found [14,15]. It is also because of such reasons that it is difficult to clearly provide a definition for a healthy microbiota.

Nevertheless, as further discussed in this review, diversity, richness and evenness in the composition of the gut bacterial community have been found to be altered or to have an effect in obese subjects [16]. Backhed *et al.* [17] observed that germ free mice remained lean when raised without microbiota despite their genetic predisposition to obesity. Interestingly, the conventionalization of mice with gut microbiota led to an improved absorption of monosaccharides. Findings suggest that microbes colonizing a mucosal surface interfere in the formation of microvasculature, which suggests microbial regulation of angiogenesis [18].

Diet is also believed to influence the composition and activity of the gut microbiota. For instance, increasing evidence shows the modulation of the gut bacterial members after following a high-fat diet, which is accompanied, in particular, with a reduction in bifidobacteria [19,20,21,22]. The high-fat diet has been shown to lead to an increase in gut permeability which influences lipopolysaccharide (LPS) plasma levels, potentially leading to inflammation [21].

Obesity could lead to an important number of metabolic diseases that include increased morbidity and mortality which implies, besides a detrimental quality of life, high health costs [1]. Therefore, a better understanding of the interaction of diet, microbiota and host are fundamental in recommending lifestyle and therapeutic approaches to tackle obesity in humans. This review focuses on the research conducted to understand the role of the gut microbiota in obesity.

## 2. Dominant Gut Microbiota in Obese Individuals

It has been suggested that the microbiota from obese individuals has an uncharacterized property that notably favors the balance towards *Firmicutes* when compared to *Bacteroidetes* [16]. Ley *et al.* [23] found a lower relative abundance of *Bacteroidetes* and a proportional increase in *Firmicutes* in obese mice. Such changes were observed to be independent from food consumption. Differences in the abundance of these two phyla were also observed in individuals under a carbohydrate or fat restricted low calorie diet [16]. The increase in *Bacteroidetes* was correlated to weight loss in these subjects. More evidence about this ratio is discussed in the section referring to “experimental evidence on the influence of gut microbiota in the development of obesity”.

Others have not detected such a relationship in the proportion or ratio of these populations and obesity, or have shown the complete opposite results [24,25,26]. Possible reasons behind the contradictory observations could be attributed to differences among the subjects studied (age, diet, geographical origin) [27,28] and the study design (whether weight loss or weight gain were studied, or a comparison between obese and lean was carried out) which has a major impact on finding differences in the data. In addition, biases inherent to the techniques used to study the composition of the microbiota can contribute to contradictory observations [29].

Molecular analysis of the gut microbiota in obese Indian individuals revealed a predominance in *Bacteroides* genus [30]. Furthermore, high archaeal densities together with high short-chain fatty acids (SCFA) levels were identified in these subjects. Therefore, as commonly referred to, this ratio is likely to be just the tip of the iceberg. Several factors may underlie the differences observed.

To begin with, different phyla (including both *Firmicutes* and *Bacteroidetes*) are composed of a wide variety of species whose role in obesity has not been evaluated and such issue remains controversial [24,31]. For instance, *Staphylococcus aureus* (*Firmicutes*) have been associated with an obese phenotype [32]. However, also *Halomonas*, and *Sphingomonas* (*Proteobacteria*) have been found in higher abundances accompanied with low *Bifidobacterium* (*Actinobacteria*) numbers when compared to lean individuals [33].

It has also been suggested that obese individuals present an increased energy uptake mediated by an important mechanism involving interspecies hydrogen (H_2_) transfer (e.g., H_2_ producing bacteria and H_2_ utilizing methanogens) [34]. Zhang *et al.* [34] proposed that such mechanism stimulates the fermentation of non-digestible carbohydrates and, therefore, an increased production of SCFA. However, as observed by Venema *et al.* [24], it remains unclear from such hypothesis how increased energy harvest can take place while there is loss of carbon.

Commensal mucosal bacteria interacting with the mucus layer may also have an effect on obesity [35]. Everard *et al.* [35] inversely correlated the abundance of *A. muciniphila* and obesity. The study clearly shows the importance of this mucin degrading bacteria in the regulation of the cross-talk between gut microbiota and the host. Their results provide good insight into the improvement in the metabolic profile in the host when this species is present as well as its role in controlling gut peptide secretion, inflammation and gut barrier function.

Recently, enrichment of the family *Christensenellaceae* has been found in lean individuals which, when transplanted to mice, have shown to promote a lean host phenotype and had an impact on the diversity of the community [36].

Finally, there is also the hypothesis that lower diversity in the gut microbiota may have an effect on satiety and eating behavior [37]. Studies have found that the gut microbial community from obese twins is less diverse when compared with lean twins [38]. In addition, antibiotic treatment studies indicate that there is an impact of long-term exposure of antibiotics on acquired obesity and weight gain [39,40,41]. In fact, antibiotics have been used in animals as growth-promoting agents prior to their ban by regulatory bodies [42].

## 3. Mechanisms by Which Microbiota May Contribute to the Development of Obesity

### 3.1. Microbiota and Adipose Tissue

Two major pathways have been suggested to explain how gut microbiota can promote fat storage.

First, by suppression of angiopoietin-like protein 4/fasting-induced adipose factor (Angptl4/Fiaf). Fiaf is produced by white and brown adipose tissue and the intestines and inhibits lipoprotein lipase (LPL) which results in the down-regulation of fatty acid oxidation in adipose and muscle tissue [2]. Therefore, LPL inhibition by Fiaf reduces fat storage, and conversely Fiaf suppression induces fat storage. Research in normal and Fiaf knockout, germ-free and conventionalized mice, showed that suppression of Fiaf by microbiota or *B. thetaiotaomicron* leads to a higher activity of LPL [17,43,44]. In response, a higher adipocyte triglyceride accumulation and cellular fat uptake was induced [17]. Despite this, evidence about the role of suppression of Fiaf by gut microbiota is still unclear, e.g., Backhed *et al.* [17] reported an increase in intestinal expression of Fiaf in germ-free mice upon conventionalization. While in the report from Fleissner *et al.* [45] intestinal expression of Fiaf was elevated in both germ-free and conventional mice with no effect on the circulating levels of the protein. Interestingly, in a study by Aronsson *et al.* [46], mice supplemented with *Lactobacillus paracasei* ssp. *paracasei* F19 showed reduction of fat storage even under a high-fat diet. In this study, circulating Fiaf levels were higher in the F19 treated group. The same alterations in Fiaf expression, but to a lesser extent, were observed in co-culture of colonic cell lines with *Bifidobacterium animalis* subsp. *lactis* Bb12 [46].

The second pathway corresponds to the influence of the gut microbiota on host energy homeostasis. From the lack of consensus in trying to correlate composition of gut microbial community and obesity, the metabolic activity of the microbiota has been suggested to play a more relevant role in the development of obesity [24]. Humans lack enzymes specialized in degrading non-digestible carbohydrates. Due to this, the bulk of dietary fiber components that pass the upper gastrointestinal tract reach the cecum and the large intestine where the anaerobic colonic microbiota is able to use these substrates for degradation through fermentation [47]. Colonic fermentation of indigestible saccharides results in the production of SCFA (primarily: acetate, propionate and butyrate [48]) mixed gases (e.g., CO_2_, CH_4_ and H_2_), formate, lactate and ethanol [49]. Since it is estimated that an average Western diet contains nearly 20–25 g of fiber per day [50] and it is suggested that SCFA products may provide daily 5%–15% of dietary energy to the host in the form of SCFA, the potential role of energy extraction by the microbiota in obesity has been of interest in recent studies.

Butyrate has been considered as an important energy source to colonocytes: it has been considered responsible for 70% of their energy needs [51,52]. However, some studies indicate that under physiological conditions, acetate may be as important as butyrate in terms of energy supply in the colon [53,54]. Due to the lack of data on SCFA production rates, it is not possible to elucidate up to what extent these SCFA are involved in human energy metabolism [51]. It is believed that the liver takes up a fraction of SCFA (approximately 30%) from the portal circulation [55,56]. However, no absolute values are available.

There is limited information about *in vivo* SCFA production. Measurement in humans is almost impossible due to ethical constraints and also because measurement of SCFA in the portal vein are not representative of those in the systemic circulation [57], or those produced in the lumen of the colon due to efficient extraction of butyrate by the colonocytes. Therefore, the investigation of colonic metabolism mainly results from fecal content analyses and *in vitro* studies [47]. However, the concentrations of SCFA measured in feces do not properly correlate with concentrations and production rate in the gut since most SCFA are rapidly assimilated by the host [47]. Approximately 95% of the SCFA produced by the microbiota are absorbed, and only 5% is estimated to be excreted in feces [58]. Despite this, the fecal analysis of metabolite production, specifically SCFA, in obese subjects evidence a higher content when compared to lean individuals, with propionate in higher proportions [26].

The study of the differences in gut metabolite concentrations in feces of obese Swiss children also showed a tendency towards a higher SCFA content in the feces from obese individuals [49]. However, no statistical difference was found in the total cumulative concentrations in the obese group when compared to overweight children. Instead, statistically differences were observed in the ratios. The authors of this study indicated that there was an overactive and aberrant metabolic activity in the gut microbiota from obese children. Yet, (i) the large standard deviation calculated for some metabolites and (ii) defining whether such activity is due to a higher production of SCFA, since a higher fecal SCFA content may also be interpreted as a reduced uptake in the colon, suggests a careful interpretation of the data.

### 3.2. Gut Microbiota and Regulation of Bile-Acid and Cholesterol Metabolism

The uptake of lipids in the small intestine is achieved by emulsification and micelle formation of fats mediated by bile salts [2]. Such bile salts are secreted as conjugates of taurine, glycine or sulfate [59] and are normally reabsorbed in the ileum [2]. A small portion of bile salts escapes the reabsorption in the small intestine and reaches the large intestine [59]. In the gut, well equipped microbiota with hydrolytic enzymes (e.g., sulfatases and β-glucuronidases) can deconjugate bile salts [59] which consequently limits the reabsorption across the mucosa [2]. When bile acids escape the enterohepatic cycle, they are excreted in the feces which stimulates more production of new bile acids at the expense of cholesterol in the liver which certainly improves blood lipid profiles [2].

It is hypothesized that the modulation of bile-acid and cholesterol metabolism by gut microbiota could also be induced by the up-regulation of transcription factors involved in processes related with nutritional-induced inflammation and lipid absorption and *de novo* lipogenesis. Furthermore, SCFA have also been found to be involved in cholesterol reduction in both humans and animals [47] (Figure 1). In particular, for propionate and acetate it has been observed that they may decrease the activity of both hepatic 3-hydroxy-3-methylglutaryl-CoA synthase and reductase (HMGCS and HMGCR, respectively) as well as acetate could increase cholesterol 7α-hydroxylase (CYP7A1) [60,61,62]. Both HMGCS and HMGCR activities are part of the initial steps for cholesterol biosynthesis [60] while CYP7A1 has been found to be involved in cholesterol-bile acid conversion [47]. Nevertheless, the impact of gut microbiota activity on the regulation of bile-acid and cholesterol metabolism remains unclear. Germ-free mice and mice treated with antibiotics present an altered metabolism of bile acids [63,64,65,66] which influences lipid absorption [67]. Still, Velagapudi *et al.* [43] observed that, in the absence of gut microbiota, lipid absorption is not altered in mice. As explained by Backhed and Crawford [67], this may be due to the fact that microbiota has been found to intervene in gut transit time which may reduce the absorption rate.

### 3.3. Microbial Metabolism of Choline

Choline has emerged as an important and previously unappreciated regulator of obesity [68]. Choline is an essential dietary nutrient involved in the mobilization of fat from the liver [69] and is an important component of cell membranes [15]. The enzymatic activity from both host and microbes may transform choline into toxic methylamines which can be further metabolized in the liver [15]. Conversion of choline into choline metabolites and the reduction of the bioavailability of choline itself in humans and mice have been associated with metabolic syndrome and plasma lipids [69] and non-alcoholic fatty liver disease [70]. Accumulating evidence shows that supplementation of choline is directly associated with the down-modulation of insulin resistance and high-fat diet-induced obesity in mice [71], and energy utilization [68], and results in improved body weight. Therefore, plasma choline metabolites have been proposed as indicators of metabolic stress. As explained by Yan *et al.* [69], alterations in liver function and circulating glucose, triglycerides, and lipoproteins are part of the metabolic stress inflicted by obesity. Betaine levels (a choline metabolite) have been inversely associated with metabolic stress in overweight subjects suggesting that betaine supplementation may alleviate metabolic disorders in subjects with a high body mass index (BMI) [69]. Such inverse association between choline and obesity suggests that the gut microbiota may negatively impact host health by reducing its availability (Figure 1).

### 3.4. Contribution to Energy Balance and Satiety

In the context of energy balance and satiety, two pathways that may play an important role in obesity represent the potential interaction between host and the microbiota. First, the regulation of adenosine monophosphate activated protein kinase (AMPK) which is a key enzyme that controls cellular energy [1]. The activation of AMPK stimulates the activity of several transcription factors that are crucial in the regulation of glucose, cholesterol and lipid metabolism, enhancing fatty acid oxidation [47]. Correspondingly, the down-regulated expression of AMPK by gut microbiota, increases adipose tissue weight by inhibiting fatty-acid oxidation which results in obesity [72,73].

The second pathway is via the activation of important G-protein coupled receptors (GPCR) that are associated with glucose and lipid metabolism [47]. SCFA produced by fermentative bacteria may play a role as signaling molecules such of GPCR which may contribute to regulation of nutrient uptake and fat deposition (Figure 1). Acetate, propionate and butyrate have been found to be ligands for GPCR41 and GPCR43 (also known as free fatty acid receptors (FFAR)-3 and FFAR-2, respectively) [74,75]. However, their affinity differs: for GPCR43: acetate = propionate > butyrate, whilst for GPCR41: butyrate = propionate > acetate [47]. The activation of GPCR41 and GPCR43 may increase gut hormones such as glucagon-like peptide-1 (GLP-1) and peptide YY (PYY) [47]. GLP-1 stimulates insulin secretion which slows down gastric emptying and promotes satiety [2]. PYY secretion decelerates intestinal transit and suppresses gut motility, and in turn, food digestion and absorption of nutrients are up-regulated [2]. Furthermore, it has been found that PYY boosts the action of insulin on glucose absorption in adipose and muscle tissue [14,47].

Strikingly, energy expenditure may be also down-regulated by SCFA via GPCR41 by alternatively activating, at the ganglionic level, the sympathetic nervous system [76]. On the other hand, leptin expression, a hormone that increases energy metabolism and inhibits the feeling of hunger, has also been found to be stimulated by GPCR41 activation [77].

GPCR may also regulate the inhibition of lipolysis by a joint activation of hormone-sensitive lipase (HSL) and adipose triglyceride lipase (ATGL) [78]. The GPCR41 ligand butyric acid has been reported to inhibit lipolysis [79], however, it has not been demonstrated that the effects are directly mediated by GPCR41 activation [78]. GPCR43 has been found to inhibit lipolysis in murine adipocytes but its expression in human subcutaneous adipose tissue (SAT) has not been detected so far [78,80].

Some studies in knockout mice have shown conflicting results: on the one hand, the activity of GPCR41 and GPCR43 receptors have been found beneficial in regard to metabolic diseases and obesity, while others have proposed that their inhibition could tackle the consequences of excess of energy intake [6]. For instance, in the study from Kimura *et al.* [81] the role of GPCR43 in fat storage was shown in mice. The authors observed that GPCR43 over expressing mice were protected against obesity whilst deficient mice were obese when fed with a normal diet. However, Bjursell *et al.* [82] showed that GPCR43 deficient mice had an increased energy expenditure, lower body fat mass and improved insulin sensitivity under a high-fat diet showing that the deficiency of GPCR43 protected from obesity.

### 3.5. Leaky-Gut and Inflammation

The intestinal epithelium of healthy individuals is indispensable for barrier function and mucosal homeostasis [3]. It acts as a gate-keeper that allows the translocation of essential macronutrients but restricts the passage of bacteria, toxic molecules and luminal antigens such as LPS [3] which may induce the production of numerous inflammatory cytokines.

LPS is continuously produced by Gram-negative bacteria in the gut and is translocated through the intestinal capillaries by a mechanism involving Toll-Like receptor 4 (TLR-4) [83]. The increase in the uptake of LPS and the permeability of the intestine induces a systemic inflammation characterized by elevated fat deposition in the liver and high circulating levels of interleukine-1 (IL-1), IL-6, plasminogen activator inhibitor-1 (PAI-1) and tumor necrosis factor alpha (TNF-α) in the blood [2]. Furthermore, LPS has been demonstrated to induce the expression of mitogen-activated protein kinases (MAPK) dependent proinflammatory cytokines and nuclear factor-κB (NF-κB) in human adipocytes [84]. Increasing plasma levels of LPS have also been associated with induction of hyperphagia and obesity [85,86]. As shown by Hotamisligil *et al.* [87], low grade inflammation is associated with leptin and insulin resistance. However, the evidence is controversial. LPS has also been found to reduce feed intake and regulate energy metabolism even in some cases independent of increasing levels of TNF-α [88,89]. Furthermore, Cani *et al.* [90] found that high-fat feeding induced a continuous intestinal absorption of LPS achieving an elevated endotoxemia and leading to weight gain independent of excessive energy intake. In their study, plasma LPS levels were suggested to depend on the high fat diet provided and suggested that such concentration is physiologically regulated by nutrients. Interestingly, increased intestinal alkaline phosphatase (IAP), an enzyme involved in LPS detoxification, has been correlated with reduced metabolic endotoxemia (defined as elevated high levels of plasma LPS) whilst a decreased activity has been associated with obesity [85,91,92].

### 3.6. Endocannabinoid System

It has been proposed that obesity may be associated with the deregulation of the endocannabinoid (eCB) system. This system regulates metabolism and appetite by the microbiota-gut-brain axis, playing a major role in energy homeostasis [3]. The eCB system is composed of locally synthesized endogenous bioactive lipids, proteins that regulate their production and degradation, and specific G protein-coupled receptors [3]. Organs that normally facilitate intake and storage of energy, such as pancreas, gut, liver, hypothalamus, adipose tissue, and muscle, synthesize eCBs in response to the demand from cell membrane phospholipids and are immediately released to target their receptors [93]. The main bioactive lipids from the eCB system are *N*-arachidonoylethanolamine (AEA) or anandamide, and 2-arachidonoylglycerol or 2-AG, whilst cannabinoid receptors 1 and 2 (CB_1_ and CB_2_, respectively) are considered to be the main receptors from the system [3,94].

The coordinated action of CB_1_ and CB_2_ in the modulation of glucose homeostasis has been suggested. Still, there is no consensus regarding the precise mechanisms involved [93]. Evidence in mice indicates that CB_1_ receptors could be selectively modulated by the microbiota, and possibly, such an effect is involved in adipogenesis, control of gut barrier function and increased food intake [3,95,96] (Figure 1). The exact role of CB_2_ receptor needs further study. However, evidence in rats indicates that CB_2_ may be involved in glucose homeostasis by improving glucose tolerance [97].

Obesity has been associated with a high eCB system tone [96]. The abnormal expression of CB_1_ and high levels of eCB in adipose tissue, brain, skeletal muscles and plasma are characteristic in obese subjects [3], whilst decreased levels of enzymes and receptors that regulate their production and degradation in other organs including heart, kidneys and stomach have been also found [98]. In addition, LPS has been found as a potent stimulator of the synthesis of eCBs [94].

All in all, the eCB system constitutes also an important target to treat obesity. However, the exact role of the receptors implicated in the system needs further clarification. For instance, the potential application of CB_1_ agonists in obese and overweight subjects has been carefully re-evaluated due to the associated psychiatric side effects which include mainly depression [3,99,100].

## 4. Experimental Evidence on the Influence of Gut Microbiota in the Development of Obesity

The gut microbiota is a dynamic community with individual needs and dependent on the host for its existence [67]. Increasing evidence from nutritional trials shows the potential link between obesity and gut microbiota. Such studies have focused efforts on determining an obese phenotype associated with specific gut microbiome and activity [1]. However, the question whether specific populations respond to diet or are responsible for weight gain remains unanswered [101]. Further investigations are needed to overcome the cause or effect dilemma.

Experimental models used to try to elucidate the role of gut microbiota in obesity include *in vitro* systems, animal models and humans. Human trials are considered as the golden standard. Still, the use of other approaches is prioritized before undertaking sometimes rather invasive and costly human interventions. Therefore, animal models have been considered as an ethically more acceptable, cheaper technique than human studies. Yet, these models do also include certain limitation to fully represent the complexity of a human being. For instance, despite the fact that it has been found that mice and humans share microbes from the main phyla, numerous bacterial genera and species from one are not detected in the other and *vice versa* [23,101]. Therefore, even though results from studies including native communities in mice are interesting, there is a high possibility that the animal’s genotype strongly influences such results [101]. In order to overcome such bias, improved animal [101] and *in vitro* models [102] in which human fecal transplantation is performed have been developed and, even with the implicit limitation of not directly studying a human being as such, they represent a viable alternative for trying to elucidate the complex role of the gut microbiota in human health.

### 4.1. Evidence from In Vitro Studies

Experiments involving *in vitro* fermentations have helped to simulate an ample number of different conditions such as age, diseases and disorders [103]. *In vitro* models closely mimicking the microbial metabolism in the human intestine can be used to get further insight into the complex mechanistic processes mediated by the gut microbiota. Hence, an *in vitro* study offers the great opportunity to examine microbe–microbe and microbe–substrate interactions in depth, by carefully controlling all variables and avoiding host derived interactions. However, findings need to be further validated in studies performed in either animals or humans.

There have been a limited number of *in vitro* reports investigating the differences of lean and obese microbiota. Few studies have provided evidence of the plasticity of the human gut microbiota in relation to dietary interventions. For instance, Payne *et al.* [91] evaluated three different Western dietary trends: high, normal and low energy reflecting obese, normal and anorectic dietary intakes in microbiota from obese and normal weight children. Their results demonstrate a metabolic adaptation of the microbiota in response to the different nutrient loads together with a reorganization of the structure of the bacterial community. Moreover, our own recent studies add to knowledge by suggesting that not all substrates are fermented in an identical manner by the gut microbiota, as clearly shown by the different measurements of SCFA and branched-chain fatty acids (BCFA) observed in lean and obese microbiota pointing to the possible implications in energy extraction if similar effects happen *in vivo* [104,105]. On the other hand, Yang *et al.* [106] observed non statistically significant differences in the microbiota activity from obese subjects when compared to lean after the *in vitro* fermentation with different dietary fibers, but small differences in propionate and butyrate production were found. Nevertheless, different *in vitro* fermentation patterns between lean and obese microbiota have not been found by others. Sarbini *et al.* [107] studied the fermentation of α-gluco-oligosaccharides and inulin and observed that they produced similar effects on bacterial population and metabolic activity in both lean and obese microbiotas. The same effect was observed by the authors when fermenting dextrans of different molecular weights [108].

Some other interesting studies such as the one from Bussolo de Souza *et al.* [105] and Condezo-Hoyos *et al.* [109] observed clear differences in lean and obese subjects at the compositional level when studying the effects of *in vitro* fermentation of fibers from cassava bagasse and apple cultivars, respectively. Cassava bagasse is a by-product from starch production and cassava flour. Bussolo de Souza *et al.* [105] observed that cassava could modulate the microbiota composition from lean and obese individuals. The obese microbiota, in particular, became similar to the lean composition after the 72 h fermentation experiments which gave a nice indication of the improvement of the community’s health. It is noteworthy that acetate production was higher in the obese microbiota, and due to the role of acetate in lipogenesis, the authors therefore speculate that such increase could be not “protective” against lipogenesis and further research is needed. Condezo-Hoyos *et al.* [109] observed an inverse trend in the proportion of *Firmicutes* and *Bacteroidetes* in feces from obese and lean. As explained by the authors, such differences may be the result of a complex and highly specific microbial ecosystem characteristic from subjects. Interestingly, these authors also found that after the administration of the fibers of different apple cultivars, the microbiota composition from obese mice tended to be similar to the lean controls.

### 4.2. Evidence from Animal Studies

Rodent models are useful since these animals can be kept in controlled environments and feeding (dosage, type) is strictly supervised [101], contrary to the conditions in human studies which lead to more variability of the results [101]. Therefore, rodent models are useful for the assessment of the role of the gut microbiota in obesity.

As observed in humans, compositional differences have also been found in the gut microbiota from lean and obese rodents. A high abundance in *Firmicutes* and low abundance in *Bacteroidetes* has been reported in obese subjects [23,110]. Yet, these proportions are inconsistent with other studies [25,26,110]. Still, a key study in which the transplantation of gut microbiota from normal to germ-free mice was performed provided crucial evidence about the potential influence of microbiota in obesity. In this study, conducted by Backhed *et al.* [17], an increase in body fat in the ex-germ-free recipients was observed without any increase in the consumption of food suggesting that the amount of energy extracted from the diet could potentially affect host homeostasis. In fact, Turnbaugh *et al.* [110] showed that changes in the relative abundance of *Firmicutes* and *Bacteroidetes* affect metabolically the function of the gut microbiota in genetically obese mice. Furthermore, a controlled diet study in gnotobiotic mice colonized with human fecal microbiota showed that after switching from a plant polysaccharide chow to a high-fat, high-sugar diet, the composition of the community drastically changed within a single day accompanied with alterations in metabolic pathways and gene expression [111]. This together represents an interesting finding of the overall effect of diet on the gut microbial community. Such finding was also supported by Hildebrandt *et al.* [112] who observed that the normal configuration of the microbiota in Resistin-like molecule-beta (RELM-β) knockout mice also changed after a high-fat diet, however, the mice were resistant to a diet-induced obesity. RELM-β acts as an effector of intestinal immune function [113].

Diet trials adding certain probiotic strains in the feeding of mice have been demonstrated to have an effect on their eating behavior and weight gain. Kondo *et al.* [114] evaluated the effect of *Bifidobacterium breve* strain B-3 in mice fed a high-fat diet. Their results suggest that this probiotic strain may importantly contribute to reduce obesity by up-regulating expression of genes involved in insulin sensitivity and fat metabolism in the gut. Furthermore, Yadav *et al.* [115] observed that feeding mice with the probiotic mixture VSL#3 resulted in the decrease of food intake by inducing GLP-1 and butyrate production. There is also evidence showing that *Bifidobacterium* spp. consumption may increase secretion of GLP-1 and PYY in the intestine [116] and reduce intestinal permeability [117] in mice. Furthermore, it has been observed that prebiotic consumption stimulates the production of GLP-2 which, at the same time, may lower plasma LPS improving mucosal barrier function by improved tight junctions [117,118].

Despite the advantages that rodent models can offer when compared to human trials, there are several disadvantages as well. Besides the differences mentioned above with respect to the gut microbiota composition found in rodents and humans, it is important to emphasize that rodents are originally granivore animals, contrary to humans (omnivores) and also practice coprophagia [12,13,119]. Fermentation in rodents primarily occur in the cecum and their digesta passage rate is faster when compared to humans [12]. Therefore, they present a lower capacity for fiber digestion [12].

Another animal model that represents an alternative to the study of the role of microbiota in obesity, is the pig. Physiologically, pigs and humans are highly similar in terms of digestive and metabolic processes [12]. Furthermore, both are colon fermenters [12]. However, there are different opinions in regard to the gut microbiota composition and its similarity to humans. Abundance of certain groups of bacteria differs. In particular, bifidobacteria in pigs are considerably lower when compared to humans (less than 1%) [120] and members of the *Clostridium coccoides–Eubacterium rectale* cluster and enterobacteriaceae are rarely found in pigs whilst the abundance of streptococci is believed to be much higher than in humans [121,122]. Yet, body fat distribution and fat cell size in pigs are comparable to humans and the propensity for pigs to a sedentary lifestyle and weight gain is also similar [13,123].

Guo *et al.* [124] and Pedersen *et al.* [125] found low proportions of *Bacteroidetes* compared to *Firmicutes* in fecal samples from obese Banna and Ossabaw mini-pigs. However, opposite results regarding such ratios were also found by Pedersen *et al.* [125] in Gottingen mini-pigs. Genetically obese pigs have also been used as models. The studies from He *et al.* [126] and Varel *et al.* [127] in pigs provide evidence that levels of microbial metabolites such as trimethylamine-*N*-oxide and choline may have a role in the development of obesity as well as a difference in the digesta passage rate among subjects.

Recently, a novel study was performed by Pedersen *et al.* [128]. The authors cloned pigs with the aim of reducing genetic influences when studying the effect of diet in obesity using this model. Yet, the inter-individual variation, in terms of microbiota composition, was not found to be reduced in the cloned pigs compared to the non-cloned pigs. Still, the relative abundance of *Firmicutes* was generally stimulated to increase over time during the diet-induced obesity intervention in both cloned and non-cloned control pigs.

### 4.3. Evidence from Human Studies

Crucial findings from *in vitro* and animal studies need to be validated ultimately through human studies. From the evidence found in pre-clinical studies, a considerable number of clinical trials have bloomed. For instance, in an effort to determine the role of gut microbiota as a regulator of obesity in humans, Ley *et al.* [16] studied obese patients who followed a therapy consisting of a fat-restricted or a carbohydrate-restricted low calorie diet. Before the diet, obese people had higher Firmicutes abundance compared to Bacteroidetes. The subjects presented significant changes in the relative abundance in the Firmicutes/Bacteroidetes ratio after following the therapy [16]. Increasing levels in Bacteroidetes were observed over time which was correlated with the percentage in body weight lost. However, the group of subjects participating in this study was small (*n* = 12). Therefore, extra caution is suggested when drawing conclusions from the study.

In addition, aberrancies in gut microbiota composition have also been associated with being overweight in a weight-gain model studied in pregnant woman. An increase of abundances in the *Bacteroides* genus and *S. aureus* was observed in these subjects [129]. Such findings suggest that some microorganisms may enhance obesity and energy storage. Indeed, Jumpertz *et al.* [130] investigated the caloric content in feces of nine obese and 12 lean individuals consuming 2400 and 3400 kcal/day diets. They found that overfeeding in lean subjects was associated with a decrease in stool energy loss. In addition, an increased energy harvest of approximately 150 kcal was also associated with a decrease in *Bacteroidetes* and a 20% increase in *Firmicutes*. Previous studies in healthy volunteers consuming diets equivalent in energy content also found a high fecal energy loss of ingested calories in stools of subjects consuming high-fiber compared to those consuming a low-fiber diet [131].

Other attempts to find the correlation between gut microbiota composition and obesity have been unsuccessful. No difference was detected in the proportions of *Bacteroidetes* between lean and obese individuals by Duncan *et al.* in a weight-loss study [25]. Instead, an increment in the *Bacteroides–Prevotella* group was observed in a similar study conducted by Nadal *et al.* [132]. Individuals after a gastric bypass have an increase in *Firmicutes* with reduced densities of archea [34]. However, no single dominant species has been identified to change in response to weight gain [110].

Studies administrating probiotics to humans also suggest an important role of the microbiota in obesity. Kadooka *et al.* [133] tested the anti-obesity effects of *Lactobacillus gasseri* SBT2055 in adults with obese tendencies. They observed an important reduction in body weight and abdominal adiposity which was not related with energy intake. In addition, a prospective investigation studying three cohorts from U.S. women and men found that yogurt consumption was the food ingredient with the highest inverse correlation in regard to weight change [134]. However, conclusions from human studies should be carefully drawn. Dietary interventions, in particular, show that there is a very strong individual variation in responses as discussed by Salonen *et al.* [135] who even suggested to stratify individuals in responders and non-responders based on the composition of their gut microbiota.

Research that may also contribute to a better understanding of the mechanisms associated with obesity is that on gastric bypass surgery. This surgery ameliorates systemic inflammation, greatly improves glycemic control, and stimulates changes in pH, gut motility, hormones secretions and bile acid flow [136,137,138]. The weight reduction achieved after this surgery has been suggested to affect the gut microbiota composition by increasing its richness in addition to beneficially influencing gene expression of white adipose tissue [137]. However, the surgery-induced modifications may also account as factors that influence the homeostasis of this community, as gastric bypass will lead to the arrival of different substrates in the colon [136].

## 5. Conclusions

The aetiology of obesity is rather complex and dependent on different factors. Alcock *et al.* [37] suggested that the human gut witnesses an evolutionary conflict between host and microbes in which the microbiota exerts a selective pressure on the host in order to increase their fitness at the expense of the host’s fitness.

We have here demonstrated what the conflicting pieces of evidence are in this field. One of the challenges that needs to be overcome for a better understanding of how microbiota affect our (human) homeostasis is to identify consistent mechanisms that are specific for the microbial activity and development in the gut that could have an effect on host obesity. This way the direct impact of gut microbial communities could be estimated better. Integration of *in vitro* data, animal models and human interventions is key for this increased understanding, and reducing the inconsistencies in the current scientific data. Thus, interventions targeting gut microbiota including antimicrobials, fecal transplantations, prebiotics and probiotics consumption, may have a major potential in modulating the composition and activity of the community as well as diet.
